# Brain Ischemia Activates β- and γ-Secretase Cleavage of Amyloid Precursor Protein: Significance in Sporadic Alzheimer’s Disease

**DOI:** 10.1007/s12035-012-8360-z

**Published:** 2012-10-19

**Authors:** Ryszard Pluta, Wanda Furmaga-Jabłońska, Ryszard Maciejewski, Marzena Ułamek-Kozioł, Mirosław Jabłoński

**Affiliations:** 1 Laboratory of Ischemic and Neurodegenerative Brain Research, Mossakowski Medical Research Centre, Polish Academy of Sciences, 02-106 Warsaw, Pawińskiego 5 Str., Poland; 2 Department of Neonate and Infant Pathology, Lublin Medical University, 20-093 Lublin, Chodzki 2 Str., Poland; 3 Department of Human Anatomy, Lublin Medical University, 20-090 Lublin, Jaczewskiego 4 Str., Poland; 4 Department of Orthopaedic and Rehabilitation, Lublin Medical University, 20-954 Lublin, Jaczewskiego 8 Str., Poland

**Keywords:** Brain ischemia, Amyloid precursor protein, β-Amyloid peptide, α-Secretase, β-Secretase, γ-Secretase, Oxidative stress, Neuronal death, Alzheimer’s disease

## Abstract

Amyloid precursor protein cleavage through β- and γ-secretases produces β-amyloid peptide, which is believed to be responsible for death of neurons and dementia in Alzheimer’s disease. Levels of β- and γ-secretase are increased in sensitive areas of the Alzheimer’s disease brain, but the mechanism of this process is unknown. In this review, we prove that brain ischemia generates expression and activity of both β- and γ-secretases. These secretases are induced in association with oxidative stress following brain ischemia. Data suggest that ischemia promotes overproduction and aggregation of β-amyloid peptide in brain, which is toxic for ischemic neuronal cells. In our review, we demonstrated the role of brain ischemia as a molecular link between the β- and the γ-secretase activities and provided a molecular explanation of the possible neuropathogenesis of sporadic Alzheimer’s disease.

## Introduction

The average life span of world societies in the last century has significantly prolonged. In the near future, a continuous increase of aged population is expected worldwide. Aging is an important risk factor for onset of ischemic stroke and Alzheimer’s disease [1–3]. Alzheimer’s disease and ischemic brain injury are characterized by widespread functional disturbances of the human brain including dementia [2, 4–8]. Some hypotheses suggest that aged neuronal cells have increased susceptibility to neuronal diseases since they are as old as the people they belong to [2, 9]. On the other hand, many of neuronal diseases can be initiated by the same mechanisms involved in their development and progression. These disorders could be progressed by abnormalities of different proteins [3, 10, 11]. Accumulation and aggregation of certain proteins could stimulate a toxic activity [12], which may stop neuronal function in damaged neurons and cause neuronal death [13]. In ischemic stroke and Alzheimer’s disease, some aberrant proteins are strongly correlated with the progression of disorder [3, 10, 11]. These proteins include different parts of amyloid precursor protein and tau protein hyperphosphorylation in brain ischemia and Alzheimer’s disease [10, 14–21]. The modified proteins and/or their products like β-amyloid peptide that is a product of parent amyloid precursor protein proteolysis can aggregate. When this process starts, proteins form pathological aggregates like amyloid plaques and neurofibrillary tangles [10, 11, 15, 16, 19–22]. Moreover, pathological aggregates can be observed in intra- and extracellular space. From medical point of view, it is important if these modified proteins are involved in neuronal dysfunction followed by neurons’ death found in these two seemingly different diseases. In any case, investigations of the mechanism connected with the onset and progression of neuronal diseases like brain ischemia and Alzheimer’s disease are of great interest in order to resolve etiopathology and, next, to develop effective treatment for both diseases.

In the review, we will deal with ischemic amyloid precursor protein metabolism by β- and γ-secretases. A possible molecular link between both secretases could be related to the onset of irreversible ischemic brain alterations [13, 23]. It is claimed that β-amyloid peptide after ischemia as well as ischemia independently induce oxidative stress that, in turn, increases β- and γ-secretase activities which further enhance β-amyloid peptide production. Molecular processes of neurons’ death are studied using experimental ischemic brain models partially because brain ischemia is a huge problem for aged society and partially because ischemic models produce reliable and reproducible data. Additionally, there are many molecular similarities and relationships between ischemic brain disorders and Alzheimer’s disease [13, 23–26]. Finally, in the human clinical setting, also Alzheimer’s disease is preceded by a significant reduction of brain blood flow [27]. Some data obtained from experimental brain ischemia investigations may apply to neuron death following ischemic brain injury. However, there should probably be many common molecular processes between ischemic neuron loss and neuron death noted in Alzheimer’s disease. In this review, we demonstrated the processes in ischemic neuron death and referred to their relation to neuron death in a brain affected by Alzheimer’s disease.

## Ischemic Oxidative Stress

**Fig. 1:**
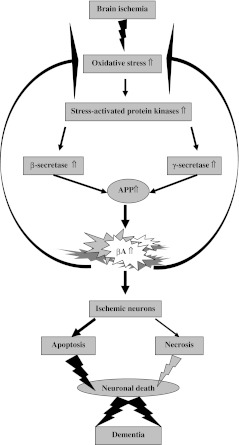
Pathogenic sequence in ischemic neuronal death through β-amyloid peptide overproduction and dementia development. *APP* amyloid precursor protein, *βA* β-amyloid peptide

Reperfusion may reverse the ischemic cascade, but at the same time, it induces a further damage [28–32]. The period of time following brain ischemia is mainly responsible for induction of oxidative stress due to formation of free radicals [28, 29, 31, 32] which culminate as harmful factors during reperfusion [33]. The reactive oxygen species, which are especially responsible for oxidative stress, include superoxide radical anion and nitric oxide. Furthermore, free radicals react with each other to form oxidant peroxynitrite. There are also other oxidant elements such as hydroxyl free radical and hydrogen peroxide [34]. Free radicals can cause membrane damage, lesions of nucleic acid, and gene damage which lead to necrotic and/or apoptotic cell death [29, 31, 32, 35] (Fig. 1).

## Amyloid Precursor Protein

In 1984, the β-amyloid peptide, the main component of the amyloid diffuse and senile plaques in brains of patients with Alzheimer’s disease, was successfully sequenced [36]. A purified protein derived from the twisted β-pleated sheet fibrils in cerebrovascular amyloidosis associated with Alzheimer’s disease has been isolated. β-Amyloid peptide is a soluble, highly aggregating small polypeptide of molecular mass 4 kDa. Moreover, Glenner and Wong [36] have claimed that β-amyloid peptide could be derived from a unique serum precursor. Next, in 1987, the discovery of the parent amyloid precursor protein initiated a huge investigation of the amyloid precursor protein-derived β-amyloid peptide [37]. There are three main isoforms of amyloid precursor protein (695, 751, and 770) derived from the alternative splicing of the amyloid precursor protein gene located in chromosome 21. Amyloid precursor protein is a type 1 integral cell surface membrane protein that resembles a signal transduction receptor [37]. Amyloid precursor protein is synthesized in the endoplasmic reticulum, modified in the Golgi apparatus, and finally transported to the cell surface *via* the secretory pathway. Amyloid precursor protein is also endocytosed from the cell surface and metabolized in the endosomal/lysosomal pathway. Proteolytic processing of amyloid precursor protein by α- or β-secretase leads to the extracellular release of soluble α-secretase-released N-terminal of amyloid precursor protein and β-secretase-released N-terminal of amyloid precursor protein, respectively. Cleavage of amyloid precursor protein on the extracellular side of the membrane by β-secretase at the N-terminal of β-amyloid peptide and on the intracellular side of the membrane by γ-secretase complex at the C-terminal of β-amyloid peptide generates β-amyloid peptide 1–42 or β-amyloid peptide 1–40 and a cytoplasmic part called an amyloid precursor protein intracellular domain. Two different forms of β-amyloid peptide are determined by γ-secretase activity. β-Amyloid peptide 1–42 was found to be the most neurotoxic form. β-Amyloid peptide is present in the blood and cerebrospinal fluid in normal individuals, which suggests that the peptide’s production is continuous in normal life [38].

Amyloid precursor protein mRNA increased twice in focal transient ischemic brain injury and remained high during 7 days following the insult [39]. In the above-mentioned ischemic injury, the Kunitz protease inhibitor-bearing isoforms were increased, but amyloid precursor protein 695 that lacks Kunitz protease inhibitor domain was decreased [40]. In focal persistent ischemia, amyloid precursor protein mRNA species that contain a Kunitz-type protease inhibitor domain were induced in the rat cortex for 21 days following the injury with maximum on the 4th day, but total amounts of amyloid precursor protein mRNA did not change [41]. During 7 days after focal ischemia, amyloid precursor protein 751 and amyloid precursor protein 770 mRNAs were induced in the ischemic area of the brain [42]. The study of focal brain ischemia in rats with ovariectomia revealed that within 1 h, there was a significant increase in amyloid precursor protein mRNA in ischemic cortex [43]. Still, estrogen treatment reduced the amyloid precursor protein mRNA overexpression in ischemic cortex [43]. This data demonstrated that estrogen may have an important role in reducing the overexpression of amyloid precursor protein mRNA following transient focal brain ischemia like in Alzheimer’s disease. Thus, these studies prove a profound effect of estrogen on ischemic brain and suggest that the hormone may be able to stop *a vicious cycle* of ischemia and neurodegenerative processes [43].

## α-Secretase

α-Secretase is cleaving amyloid precursor protein in the center of the β-amyloid peptide, and this pathway is non-amyloidogenic. This process increases extracellular secretion of the soluble α-secretase-released N-terminal of amyloid precursor protein domain which stops production of β-amyloid peptide and prevents its deposition in plaques. On the other hand, a decrease in α-secretase function contributes to the development of amyloid plaques and Alzheimer’s disease. Additionally, α-secretase is involved in inflammation which is supported by expression of α-secretase by astrocytes. α-Secretase mRNA in the hippocampus was downregulated, and the activity of α-secretase was decreased after chronic brain hypoperfusion [44]. α-Secretase decrease was noted in animals after ischemic brain injury, too [45]. That may subsequently result in an accumulation of amyloid precursor protein in the ischemic brain and then activate the amyloidogenic pathway cleaving amyloid precursor protein. Finally, the formation of β-amyloid peptide in postischemia increases and impairs the memory [44].

## β-Secretase

β-Secretase was found as a type 1 transmembrane protease. β-Secretase cleaves amyloid precursor protein at the N-terminal position of β-amyloid peptide, and this pathway is amyloidogenic. This protease is expressed in neuronal and glial cells. β-Secretase level, activity, and its product are increased in platelets in Alzheimer’s disease individuals [46]. Huge platelets pathology was observed in the ischemic brain, too [47]. In addition to this, changes in β-secretase expression and activity indicate a transcriptional and/or translational control of β-secretase expression in brain [48]. β-Secretase and amyloid precursor proteins follow similar trafficking routs and colocalize within endosomes, thus providing for optimal β-secretase activity. This is followed by a significant increase of intracellular β-amyloid peptide as well as by functional and morphological signs of apoptotic neuronal death [49] (Fig. 1). The amyloidogenic processing of the amyloid precursor protein by β-secretase is important to β-amyloid peptide plaque development in the ischemic brain [14–16, 19, 21, 50] and Alzheimer’s disease [10]. Current data showed that an experimental brain ischemia generates the overexpression, production, and activity of Alzheimer’s disease β-secretase [51–54] (Fig. 1). Other study showed for the first time the alteration in mRNA expression of three amyloid precursor protein metabolism-related genes: β-secretase (BACE1), cathepsin B, and glutaminyl cyclase mRNA, whose expression increased in the hippocampus and cortex quickly following instant recirculation [55]. One month after, BACE1 mRNA level dropped subsequently but was still above the control level during the whole period of observation. Another data have demonstrated that full-length presenilin interacts with immature β-secretase. This observation implies that presenilin regulates β-secretase activity via direct interaction and facilitates trafficking of β-secretase to different compartments of cells [56].

## γ-Secretase

γ-Secretase is an intramembranous protease complex of four essential membrane proteins called aph-1, pen-2, nicastrin, and presenilin. Aph-1, pen-2, and nicastrin function as transporters of γ-secretase, and they identify protease substrates. Presenilin represents in complex catalytically active component of the γ-secretase. γ-Secretase cleaves many type-1 membrane proteins including the Notch receptor [57], the amyloid precursor protein [49], and low-density lipoprotein receptor-related protein [58]. Interest in γ-secretase comes in part from the fact that this enzymatic complex is responsible for the cleavage of amyloid precursor protein that generates the β-amyloid peptide, one of key components of amyloid plaques in Alzheimer’s disease [10] and in ischemic human brain injury [15, 16, 19, 21, 50]. Presenilin is implicated in different processes including influence on calcium and glutamate homeostasis and cell death [23]. Recent data have shown that microglial cells and astrocytes presented overexpression of presenilin and nicastrin after brain injury [59]. Presenilin influences inflammatory processes, probably independently from β-amyloid peptide in ischemic brain.

The protein products of the genes on chromosomes 14 and 1 are presenilin 1 and presenilin 2, respectively. The first study of presenilin 1 mRNA overexpression in the gerbil ischemic hippocampus was performed by Tanimukai et al. [60]. Postischemic selective induction of presenilin 1 gene in neurons of CA3 area and dentate gyrus was observed, which might be related to the resistant areas after ischemia. In this investigation, presenilin 1 mRNA was induced by 3 days. These data suggest that overexpression of presenilin 1 mRNA may be associated with some response of ischemically injured neurons. In next research, the expression of presenilins mRNA was investigated in the rat ischemic hippocampus, cortex, striatum, and cerebellum [61]. The increased levels of presenilins mRNA exhibited the maximal value in the hippocampus and cortex sectors of massive plaque formation in Alzheimer’s disease brain. But the presenilin 1 and 2 genes expression in cerebellum and striatum displayed no significant increase, and that correlated very well with areas unaffected by Alzheimer’s disease pathology. The overexpressions were larger on the contralateral side to the focal brain ischemic injury. This significant difference may reflect a loss of brain cells expressing presenilins genes on the ipsilateral side. Staining of presenilin was more marked in glial than in neuronal cells and in a trace of the ischemic pyramidal cells of hippocampus [62]. Presenilin is involved in the amyloidogenic processing of amyloid precursor protein to produce β-amyloid peptide through the γ-secretase complex (Fig. 1). It was noted that animals with brain ischemia treated by γ-secretase inhibitors demonstrated reduced damage of brain and improved functional recovery [57]. Recently, it has been found that focal brain ischemia induces an increase of γ-secretase activity in the ischemic hemisphere [58]. The understanding of the mechanism by which γ-secretase recognizes and cleaves different proteins is of great importance to clarify the activity of γ-secretase and its role in ischemic brain and Alzheimer’s disease degeneration.

## Link Between β- and γ-Secretases

**Fig. 2:**
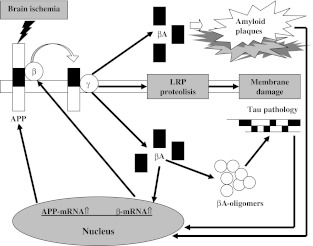
Amyloidogenic cleavage of amyloid precursor protein with its consequences following ischemic brain injury. *APP* amyloid precursor protein, *β* β-secretase, *γ* γ-secretase, *βA* β-amyloid peptide, *LRP* low-density lipoprotein receptor-related protein

Current data imply a correlation between triggered oxidative stress and increased γ-secretase cleavage of amyloid precursor protein [63, 64]. Oxidative stress can influence β- and γ-secretase activities [23, 49, 50, 64], so we can speculate that ischemic oxidative stress is the molecular link between γ- and β-secretases, and as a consequence, the activities of both proteases are linked to each other (Figs. 1 and 2). It was noted before that ischemic oxidative stress increases presenilin expression [60, 61], staining [62], and γ-secretase activity [58] in ischemic brain. Parallel β-amyloid peptide mediates oxidative stress [12] itself that, in turn, increases the function of the γ- and β-secretases, and as result, it enhances β-amyloid peptide formation (Fig. 1). The activation of β-secretase requires γ-secretase cleavage of amyloid precursor protein and is proportional to the level of β-amyloid peptide produced [49] (Figs. 1 and 2). Finally, a soluble β-amyloid peptide form causes necrotic and apoptotic neuronal death [49] (Fig. 1). Nevertheless, fibrillar β-amyloid peptide form can generate β-secretase overexpression and increase activity that results in amyloidogenic cleavage of amyloid precursor protein. Ischemic data suggest that there, probably, is a positive feedback loop between the β-secretase and the γ-secretase processing of amyloid precursor protein [23, 50], stimulated by the release of the different forms of β-amyloid peptide, which finally act as important uninvestigated signaling substances. Evidence that supports the above-mentioned material from experimental, global, and focal brain ischemia which are followed by recirculation demonstrated strong, abnormal brain staining to the N-terminal of amyloid precursor protein and to the β-amyloid peptide and to the C-terminal of amyloid precursor protein, too. There was noted not only intracellular staining [14, 22, 65–80] but also extracellular one [14, 71, 74, 77, 80, 81].

Another data have exhibited that full-length presenilin interacts with immature β-secretase. This evidence suggests that presenilin regulates β-secretase activity via direct interaction and facilitates trafficking of β-secretase to different compartments of cells [56]. Such evidence provides a molecular explanation for the role of ischemic oxidative stress in sporadic Alzheimer’s disease development (Fig. 1).

## Ischemic Cell Death

Oxidative stress becomes pathological in the brain when the formation of reactive oxygen species overwhelms the ability of the endogenous antioxidant mechanisms to neutralize excess of reactive oxygen species, which finally leads to cell damage/death [12, 29, 31, 32, 35, 82]. Increased generation of reactive oxygen species results in different pathological changes including cleavage of DNA and damage of membrane lipids (Fig. 2). Additionally, reactive oxygen species can block mitochondrial respiratory chain [34]. As a result of the above phenomenon, oxidative stress stimulates mitochondria to develop transition pore which makes mitochondria release apoptosis-related protein following ischemic brain injury [34, 83, 84]. Moreover, oxidative injury to endoplasmic reticulum may be involved in neuron death through apoptotic machinery following brain ischemia, too [85] (Fig. 1). Reactive oxygen species activate different interlinked signaling pathways which can be involved in ischemic decisions about cell survival/death such as the stress-activated protein kinases, c-Jun N-terminal kinases, p38 mitogen-activated protein kinases, extracellular signal-regulated kinases, and Akt pathways [86–88]. The p38 mitogen-activated protein kinase promotes the stabilization and enhanced translation of mRNAs encoding proinflammatory proteins [89]. Besides, reactive oxygen species can activate transcription factors, especially the nuclear factor-kappa B that regulates cell survival/death by proinflammatory cytokines [33]. Oxidative injury does not occur in isolation after ischemic brain injury but involve in the complex interactions between excitotoxicity, inflammation, overproduction of β-amyloid peptide, and apoptosis [90]. After brain ischemia, phosphorylated p38 mitogen-activated protein kinase was noted in hippocampal neurons [87] and microglia [91], suggesting its role in the endogenous inflammatory response. Furthermore, p38 mitogen-activated protein kinase inhibitors have been proved to decrease brain injury and improve neurological recovery after brain ischemia and reduce ischemic inducted cytokine overexpression [92]. On the basis of the data provided, it can be concluded that ischemia contributes to cell death caused by β-amyloid peptide overproduction (Fig. 1). This process is supported by a downregulation of α-secretase and upregulation of β- and γ-secretases [23, 50]. Overexpression of β- and γ-secretases following brain ischemia is strongly associated with an increase of stress-activated protein kinases activities. As a result, we can observe β-amyloid peptide-dependent massive neuronal death after ischemic brain injury. This suggests that brain ischemia leads to a shift in amyloid precursor protein processing from the α-secretase to β- and γ-secretase pathways with β-amyloid peptide overproduction and accumulation in extracellular space (Fig. 1). Richness of data implicates the extracellular accumulation of β-amyloid peptide in the brain as one of the important triggers of inflammation [23]. On the other hand, a receptor for advanced glycation end products was shown to be overexpressed by several folds in injured microglia and neuronal cells and endothelium [21, 93–96]. Anyway, β-amyloid peptide activates microglia cells by binding to the receptor for advanced glycation end products [97] and to scavenger receptor [98]. A receptor for advanced glycation end products binding β-amyloid peptide on neuronal cells can kill them directly by formatting inflammatory factors or indirectly by activating microglia cells [93, 97]. In contrast, ischemia also induces the production of intracellular β-amyloid peptide what was shown by immunocytochemical investigation [14, 22, 74, 77, 80]. Oligomeric β-amyloid peptide is toxic [12] and initiates a series of events in ischemic brain including the hyperphosphorylation of tau protein that results in severe neurons [11, 20], microglia [99], and oligodendrocytes [100] pathology. In addition to this, a recent research showed that, in the early stages of amyloid pathology, microplaques develop rapidly and locally, which could damage neighboring axons and dendrites within a few days [101], which eventually causes retrograde neuronal death. So far, it has been claimed that the interaction between newly formed amyloid microplaques and microglia shows that, unless further activated, microglia clear plaques unsuccessfully. However, they may restrict their growth leading to their steady size after initial formation [101].

Recently, there has been proposed a mechanism in which ischemic neuronal death is the result of a cell signaling cascade initiated by the shedding of low-density lipoprotein receptor-related protein ectodomain [102] (Fig. 2). This is followed by γ-secretase-mediated cleavage of low-density lipoprotein receptor-related protein transmembrane domain and nuclear translocation of low-density lipoprotein receptor-related protein intracellular domain [58] (Fig. 1). Altogether, these data indicate that regulated intramembrane proteolysis of low-density lipoprotein receptor-related protein is a novel pathway for ischemic neuronal death (Fig. 2) and a potential target for the therapy of ischemic stroke in human clinic. Several lines of evidence support the key role of β-amyloid peptide in the neuropathogenesis of brain ischemia like in Alzheimer’s disease [25, 103].

## Ischemia and Pathogenesis of Sporadic Alzheimer’s Disease

Some evidence suggests that the activity of β- and γ-secretases is upregulated in the brain after ischemia [23, 50, 53, 58]. As a result of the above phenomenon, brain ischemia triggers generation of β-amyloid peptide from parent overexpressed amyloid precursor protein. Probably, oxidative stress is involved in this phenomenon since this is a reperfusion-dependent process. Oxidative stress and β-amyloid peptide generation are reciprocally linked to each other because β-amyloid peptide accumulations have been proved to stimulate oxidative stress [12, 104]. Additionally, oxidative stress increases synthesis of β-amyloid peptide [105]. We hypothesized that ischemic cascade, including brain ischemia as trigger, oxidative stress, stress-activated protein kinases, and β- and γ-secretases, increased activity, and overproduction of β-amyloid peptide finally leads to neuronal cell death through necrosis and apoptosis (Fig. 1). These activities are followed by increased staining of intra- and extracellular β-amyloid peptide [14, 74, 77, 80] and by neuropathological signs of necrotic and apoptotic neuron death [31, 32]. These data support the idea that ischemic overproduction of β-amyloid peptide dependent on β- and γ-secretase-upregulated activities triggered by ischemic oxidative injury is implicated in sporadic Alzheimer’s disease etiology.

In this review, three implications of ischemic oxidative stress to sporadic pathway of Alzheimer’s disease development have been analyzed. First, data suggest that ischemic oxidative stress intensifies β- and γ-secretase activities, which result in increasing β-amyloid peptide formation. Secondly, evidence demonstrated that the existence of a positive feedback loop, where increased γ-secretase activity enhances β-secretase expression and activity, is mediated partially by the generation of β-amyloid peptide which acts as a signaling molecule. Thirdly, this review shows that the activation of the positive feedback loop by β- and γ-secretases needs the stress-activated protein kinases signaling cascade which is generated in Alzheimer’s disease apoptosis. In animals, ischemia increases β-amyloid peptide formation [14, 22, 74, 77, 80], accumulation of hyperphosphorylated tau protein, and filament generation similar to the one present in human Alzheimer’s disease brain [17, 18, 20, 106, 107]. This implies that ischemic episodes may develop neuropathological alterations similar to those seen in Alzheimer’s disease patients [24, 103].

## Conclusions

Nowadays, there is a large body of direct evidence linking brain ischemia to Alzheimer’s disease [25, 103]. This association manifests neuropathologically by the presence of neuritic plaques, tangles, inflammation, massive neuronal death, and dementia in ischemic brain. It is considered that β-amyloid peptide that is the product of β- and γ-secretases proteolysis of amyloid precursor protein could be the important factor of progressing ischemic pathology [13] and, therefore, influencing the role of secretases involved in its formation. According to recent data, these proteases can probably be regulated by inflammation with oxidative stress, which is developing after brain ischemia. We have hypothesized that the inflammatory response generated as a result of ischemia triggers the Alzheimer’s disease changes [108]. Ischemic brain alterations have been proved to increase β-secretase [53] activity in parenchyma with a concomitant overexpression of amyloid precursor protein and, subsequently, β-amyloid peptide production. It is suggested that this action should be mediated by binding of hypoxia-inducible factor 1 alpha to the promoter area of β-secretase mRNA resulting in increased levels of β-secretase enzyme in parenchyma [109]. Additionally, current data showed that brain damage increases the expression of presenilin 1 and nicastrin in glial cells, both elements of the γ-secretase complex [58, 59]. Another study revealed that tau blocks transport of amyloid precursor protein from the neuronal cell body into axons, and dendrites of neuronal cells cause amyloid precursor deposition in the neuronal body [110].

Contemporary investigations demonstrated that, following brain ischemia, hyperphosphorylated tau protein accumulates in cortical neuronal cells and colocalizes with signs of apoptosis. This mechanism may be involved in the pathogenesis in ischemic brain degeneration (Fig. 2). The above data indicate that ischemic neuronal apoptosis is associated with tau protein hyperphosphorylation [17, 20]. Wen et al. [17, 20] reported that neurofibrillary tangle-like tauopathy development in the brain in adult female is involved in irreversible ischemic rat brain injury. These data provide a neuropathological basis for the development of Alzheimer-type dementia in postischemic brains [7, 18, 111].

To conclude, in this review, we have tried to give a perspective on the wide variety of interactions between ischemic brain factors and amyloid precursor protein secretases. On the one hand, ischemic oxidative stress is able to increase the levels of all amyloidogenic secretases. On the other hand, some secretases, such as β- and γ-secretases, regulate the level of β-amyloid peptide. Bringing all these data together, it is clear that the association between ischemic brain and Alzheimer’s disease as suggested by the wealth of clinical and experimental results is based on a series of complex molecular interactions that we are only just beginning to understand in detail.
